# Infusing Technology Into Perinatal Home Visitation in the United States for Women Experiencing Intimate Partner Violence: Exploring the Interpretive Flexibility of an mHealth Intervention

**DOI:** 10.2196/jmir.6251

**Published:** 2016-11-17

**Authors:** Loraine J Bacchus, Linda Bullock, Phyllis Sharps, Camille Burnett, Donna L Schminkey, Ana Maria Buller, Jacquelyn Campbell

**Affiliations:** ^1^ Department of Global Health and Development London School of Hygiene & Tropical Medicine London United Kingdom; ^2^ School of Nursing University of Virginia Charlottesville, VA United States; ^3^ School of Nursing Johns Hopkins University Baltimore, MD United States

**Keywords:** intimate partner violence, screening, home visitation, nurses, mhealth, intervention, technology, interpretive flexibility

## Abstract

**Background:**

Intimate partner violence (IPV) is common during pregnancy and the postpartum. Perinatal home visitation provides favorable conditions in which to identify and support women affected by IPV. However, the use of mHealth for delivering IPV interventions in perinatal home visiting has not been explored.

**Objective:**

Our objective was to conduct a nested qualitative interpretive study to explore perinatal home visitors’ and women’s perceptions and experiences of the Domestic Violence Enhanced Home Visitation Program (DOVE) using mHealth technology (ie, a computer tablet) or a home visitor-administered, paper-based method.

**Methods:**

We used purposive sampling, using maximum variation, to select women enrolled in a US-based randomized controlled trial of the DOVE intervention for semistructured interviews. Selection criteria were discussed with the trial research team and 32 women were invited to participate. We invited 45 home visitors at the 8 study sites to participate in an interview, along with the 2 DOVE program designers. Nonparticipant observations of home visits with trial participants who chose not to participate in semistructured interviews were undertaken.

**Results:**

We conducted 51 interviews with 26 women, 23 home visiting staff at rural and urban sites, and the 2 DOVE program designers. We conducted 4 nonparticipant observations. Among 18 IPV-positive women, 7 used the computer tablet and 11 used the home visitor method. Among 8 IPV-negative women, 7 used the home visitor method. The computer tablet was viewed as a safe and confidential way for abused women to disclose their experiences without fear of being judged. The meanings that the DOVE technology held for home visitors and women led to its construction as either an impersonal artifact that was an impediment to discussion of IPV or a conduit through which interpersonal connection could be deepened, thereby facilitating discussion about IPV. Women’s and home visitors’ comfort with either method of screening was positively influenced by factors such as having established trust and rapport, as well as good interpersonal communication. The technology helped reduce the anticipated stigma associated with disclosing abuse. The didactic intervention video was a limiting feature, as the content could not be tailored to accommodate the fluidity of women’s circumstances.

**Conclusions:**

Users and developers of technology-based IPV interventions need to consider the context in which they are being embedded and the importance of the patient-provider relationship in promoting behavior change in order to realize the full benefits. An mHealth approach can and should be used as a tool for initiating discussion about IPV, assisting women in enhancing their safety and exploring help-seeking options. However, training for home visitors is required to ensure that a computer tablet is used to complement and enhance the therapeutic relationship.

**ClinicalTrial:**

Clinicaltrials.gov NCT01688427; https://clinicaltrials.gov/ct2/show/NCT01688427 (Archived by WebCite at http://www.webcitation.org/6limSWdZP)

## Introduction

Intimate partner violence (IPV) is recognized globally as a serious public health issue, with 1 in 3 women having experienced either physical or sexual violence from a partner [1]. Due to the adverse health outcomes, health care providers frequently, but often unknowingly, come into contact with women affected by IPV, thus providing opportunities for screening and intervention [2,3]. Debates about universal screening for IPV have resulted in conflicting recommendations for health care providers. The World Health Organization advocates symptom-prompted inquiry for IPV, while the US Preventive Services Task Force recommends universal IPV screening of women of childbearing age [4,5]. Nevertheless, research shows that women want health care providers to listen, provide sensitive and nonjudgmental inquiry about their needs, respect their wishes, and facilitate access to services [6].

Pregnancy and the postpartum can be a time of increased vulnerability for abused women because of changes in women’s physical, social, emotional, and economic needs [7]. A review of studies found that 1% to 30% of pregnant women experienced physical violence during pregnancy, with most estimates being between 3% and 11% [8]. Higher rates of IPV have been reported during the postpartum period compared with during pregnancy [9]. In the United States, perinatal home visitation is a community health strategy that has been shown to improve outcomes for families and prevent child maltreatment and neglect [10]. The long-term nature of the relationship between the home visitor and the family provides favorable conditions in which to screen women for IPV and provide support. The home visitor is able to observe aspects of family life that are not discernible in a clinical setting, which may offer clues to the presence of abuse.

However, assessing for IPV in the home is as challenging as in a clinic setting [11]. Barriers to screening include provider discomfort with IPV questioning, fear of offending women, lack of training, confidentiality issues, and time restrictions [12-14]. Mobile health technology (mHealth) such as mobile phones and other wireless computing devices may offer a solution to some of these problems, as they can allow for more confidentiality, may be beneficial for women who are unwilling to disclose abuse to a health professional, and may help to standardize the way IPV assessments and interventions are delivered [15].

Greenhalgh and Swinglehurst contended that technology in health care is often introduced with expectations of higher quality, more efficient and safer care, and empowerment for patients [16]. Empowerment is a major goal of IPV interventions, and there is some evidence that such approaches can be embedded within technology. A study using a Web-based IPV intervention (Internet Resource for Intervention and Safety, IRIS) conducted in the United States drew on Dutton’s theoretical framework of empowerment [17] by creating a safety decision aid that enhanced women’s choice making and reduced their decisional conflict [18]. Adapted versions of the safety decision aid are being tested in Australia and New Zealand [19,20]. Additionally, studies conducted in clinical settings in North America found significantly higher rates of disclosure of abuse using computerized screening than using health care provider screening methods [15,21]. However, to our knowledge, the use of mHealth IPV screening in perinatal home visiting has not been investigated. This innovative approach warrants further exploration of how home visitors and women integrate technology-based IPV interventions in a nonclinical context, where the development of a trusting relationship provides the foundation for the care provided.

The technology literature reveals polarized positions regarding the relationship between technological artifacts and human practices. This has resulted in commentators focusing on technology as either a causal agent of change, whereby human behavior and organizations are influenced by technology (technological determinism), or constructed and interpreted flexibly through human agency (social constructivism) [22]. The inherent interpretive flexibility of technology refers to its capacity to sustain the divergent opinions of different user groups, both during its construction and in the way that it is eventually used. In using technology, users are influenced by individual and social factors that lead them to interpret and appropriate it in different ways. This is evident in empirical evidence that the application of identical technologies in similar organizations can have an impact in different ways [22,23]. However, researchers have highlighted that the interpretive flexibility of technology is not limitless, that the composition of technical objects can constrain the ways in which technology can be interpreted [23,24], and that the extreme positions capturing the relationship between technology and humans present a false dichotomy. There is growing consensus among researchers that technology is both shaping of and shaped by its social context [22].

**Figure 1:**
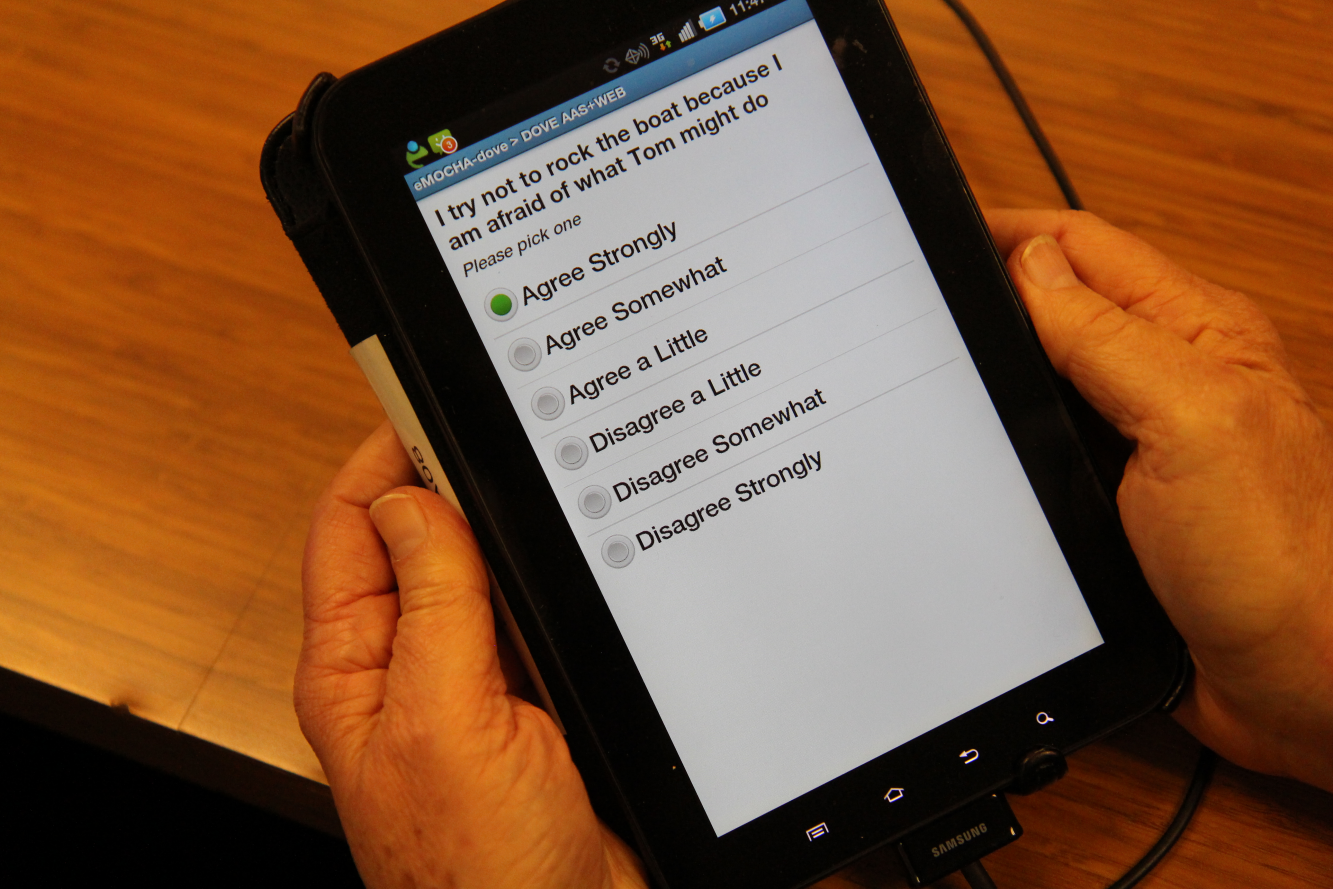
Domestic Violence Enhanced Home Visitation Program (DOVE) screenshot of one item from the Women's Experience with Battering scale. Image credit: University of Virginia, School of Nursing 2016.

**Figure 2:**
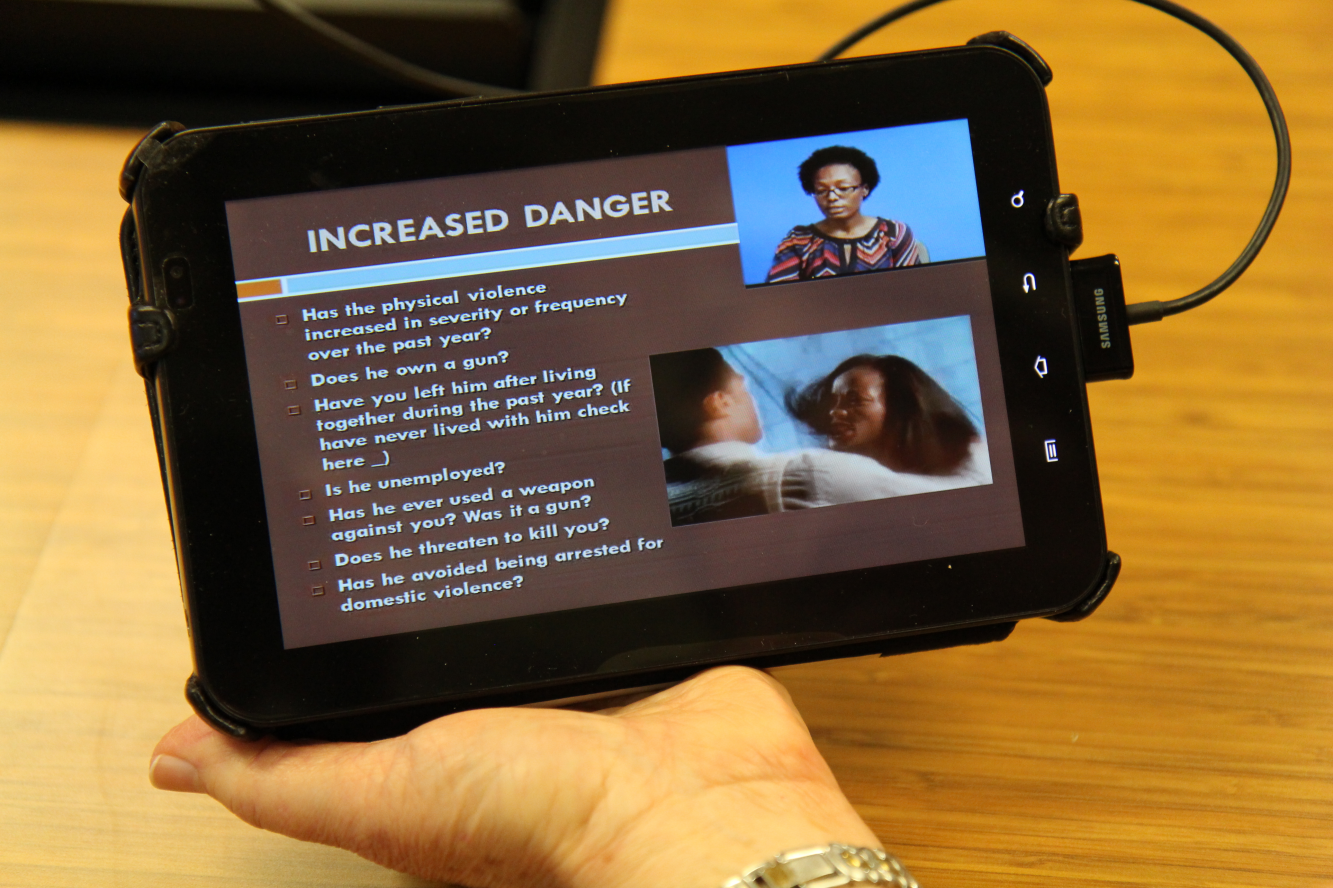
Domestic Violence Enhanced Home Visitation Program (DOVE) screenshot of the Danger Assessment scale. Image credit: University of Virginia, School of Nursing 2016.

This study explored the relationship between technology and humans in relation to the Domestic Violence Enhanced Home Visitation Program (DOVE), an empowerment intervention to prevent IPV during pregnancy, which has been integrated into perinatal home visiting programs in the United States [25]. A US multisite randomized controlled trial based in Virginia, Missouri, and Maryland (Baltimore) compared a home visitor-led method of screening for IPV and delivering an empowerment intervention, with an mHealth version of DOVE. In the home visitor method, women were screened for IPV with paper versions of the Abuse Assessment Screen [26] and Women’s Experience with Battering scale [27]. Women who scored positive for IPV in the year before the current pregnancy were eligible to receive the empowerment intervention, a home visitor-led discussion of the DOVE pamphlet, which was offered on 6 occasions at 1-month intervals. The pamphlet included information on the definition and types of IPV, the cycle of abuse, IPV during pregnancy and the health consequences, assessment of the risk factors for homicide using the Danger Assessment scale [28], safety planning, and information about community resources. In the second method, the mHealth platform electronic Mobile Open-source Comprehensive Health Application (eMOCHA) developed by Johns Hopkins Center for Clinical Global Health Education was used to deliver the same materials via mHealth, except for the safety plan that the home visitor developed with the woman. A prerecorded video presented information contained in the DOVE pamphlet. Figure 1 presents a screen shot of one of the items on the Women’s Experience with Battering scale and Figure 2 presents a screen shot of the Danger Assessment scale.

Home visitors were provided with training in IPV and the DOVE protocol using both methods. Women who were pregnant or up to 3 months postpartum were introduced to DOVE at a safe and appropriate time, which was left to the discretion of the home visitor. Women assigned to the computer tablet were free to complete the screening questions alone and were not obliged to discuss their answers with their home visitor immediately. However, the research team informed home visitors if a woman had experienced IPV in the year before her current pregnancy and therefore was eligible to receive the DOVE intervention, which was offered at a follow-up visit. Women were provided with study information and gave consent to using the computer tablet, which then randomly assigned them to the home visitor or computer tablet method. All materials were available in English and Spanish. The computer tablet remained in the possession of the home visitor and was never left in women's homes.

The aim of this study was to explore perinatal home visitors’ and women’s experiences of screening for IPV and receiving DOVE in the form of either mHealth technology (ie, a computer tablet) or a home visitor-led method. Furthermore, we aimed to understand how their perceptions of the technology resulted in differences in the outcomes of its use.

## Methods

The nested qualitative interpretive study was conceived after design and implementation of the DOVE trial. The study is underpinned by an interpretivist paradigm, which posits that reality is multiple and relative [29,30]. Using this paradigm permitted us to argue that women’s understandings and experiences of DOVE were diverse and socially constructed, influenced by factors such as the interactions generated within the context, values, culture, and time. According to Greenhalgh and Swinglehurst, interpretivists view technological interventions as part of complex social practices involving different actors, which must be understood in terms of the interpretation of the social practices that the actors bring to using technology [16]. As such, it was important to understand the care setting in which the DOVE technology was used, the meaning that it held for different users, how it affected the home visitor-client relationship, and the diverse ways in which it was interpreted and used in context.

### Data Collection Methods

#### Interviews

Between November 2013 and August 2014, the first author (LJB) conducted semistructured interviews with perinatal home visitors and women enrolled in DOVE. Interviews lasted between 1 and 2 hours and used a topic guide that explored a wide range of areas. However, this paper presents findings related to (1) screening for IPV at home using either method, (2) safety and confidentiality, and (3) aspects of the home visitor-client relationship that affected discussion about IPV and how the technology transformed this relationship through different interpretations of the technology’s use. Interviews with women and home visitors continued until data saturation was achieved.

We also interviewed the 2 program designers of DOVE who were responsible for working with the research team to create an mHealth version of the DOVE intervention, which would also capture research data for the trial. The program designers provided support for technical problems in the field and for making adaptations to the program. The interview provided contextual information about assumptions underpinning the design; computer tablet features and usability; technical difficulties experienced by end users and how these were resolved; and views on the potential for future adaptation.

#### Nonparticipant Observations

In June 2014, the first author (LJB) undertook nonparticipant observations of home visits at one rural site to gain insight into the context of care, including the physical environment, routine aspects of perinatal home visiting care, and home visitor-client interactions and behavior. Condensed field notes were written immediately after each observation and an expanded account was written at the end of the day [31]. Observation fieldwork notes included descriptive data along with the researcher’s own reflections and interpretations.

### Study Procedures and Ethics

We used purposeful sampling using maximum variation to select women based on different factors that might influence their experience of DOVE, which would provide “information rich cases for in-depth study” [32]. This was discussed in advance with the trial research team, which led to sampling women in rural versus urban locations; women who used the home visitor paper method versus the computer tablet; women who had experienced IPV versus women who had not; and age (to include younger and older women). At a later stage of the study, we attempted to sample Spanish-speaking women, as the interim results from the screening (by either method) showed that many of these women were not disclosing experiences of IPV.

The trial coordinator provided a list of 47 women enrolled to the DOVE trial who had consented to participating in a qualitative interview, along with information on the above factors. Of these, 32 women were invited to participate (of whom 6 declined) and 15 could not be contacted for various reasons (ie, telephone number no longer in use, a male constantly answering the phone, or a woman not returning messages). Interviews with women took place in their homes if it was safe to do so, or away from the home in the researcher’s car. We invited 45 home visitors at the 8 study sites to participate in an interview, which was conducted at their office. The 2 designers of the DOVE computer tablet were interviewed together via Skype.

We obtained written consent from all participants, who received a gift voucher (US $15 for home visitors and program designers, and US $30 for women) for their assistance. The study was approved by the University of Virginia Institutional Review Board for Social and Behavioral Sciences (2011-0243-00) and (2014-0075-00) and the European Union ethics review panel (February 13, 2013; proposal number 329765).

### Analysis

Interviews were digitally recorded and transcribed verbatim. Field notes from observations were typed up. We used NVivo 10 software (QSR International Pty Ltd) to facilitate data analysis. Thematic analysis was used to identify, analyze, and report on patterns within the data [33]. The initial coding framework in NVivo was guided by the interview schedule themes and was deductive. Deeper exploration and interrogation of the data was inductive, allowing additional themes and their subcategories to emerge [34]. To ensure consistency in coding, 3 women’s interviews and 2 home visitor interviews were coded by CB, DLS, and AMB using the framework, and discrepancies were discussed [34]. As a further check for consistency, LB reviewed a range of quotes representing each theme in the first draft of this paper. Interviews conducted in Spanish were translated into English, and the recording and transcript were compared for accuracy by a Spanish-speaking research nurse. In the quotes presented, IPV *+* refers to women who disclosed IPV in the year prior to their current pregnancy and IPV– refers to women who did not disclose IPV in the year prior to their current pregnancy in response to screening during the DOVE trial. During the analysis, data from the different sources were compared and integrated in relation to the key themes. Quotes presented are taken from the interviews, and data from observations are indicated throughout the text. Pseudonyms are used in the presentation of the results.

## Results

### Participant Characteristics

We interviewed 51 participants (23 home visiting staff, 26 women, and 2 DOVE computer program designers) and conducted 4 nonparticipant observations. Table 1 presents the sociodemographic characteristics of 26 women interviewed who were enrolled in DOVE.

**Table 1:** Sociodemographic characteristics of women enrolled in the Domestic Violence Enhanced Home Visitation Program (DOVE) (N=26).

Sociodemographic variables		n	%
**Age range (years)**			
	16-19	4	15
	20-23	11	42
	24-27	7	27
	28-35	4	15
**Ethnicity**			
	White	12	46
	African/African American/black	8	31
	Mixed ethnic origin	4	15
	Not reported	2	8
**Language**			
	English	23	88
	Spanish	3	12
**Location**			
	Urban	7	27
	Rural	19	73
**Marital status**			
	Married	1	4
	Single	17	65
	Partnered, not married	8	31
**Educational level attained**			
	7th to 9th grade	1	4
	10th to 12th grade	7	27
	High school graduate/GED^a^	7	27
	Some college or trade school	10	38
	College graduate	1	4
**Number of live births at interview**			
	1	17	65
	2	4	15
	3	3	12
	4	1	4
	5	1	4
**Number of partners in the year before current pregnancy**			
	1	16	62
	2	6	23
	>2	4	15
**IPV^b^** **abuse status from screening**			
	IPV in year before current pregnancy	18	69
	No IPV in year before current pregnancy^c^	8	31
**DOVE method**			
	Home visitor, paper based	18	65
	Computer tablet	8	35

^a^GED: General Education Development.

^b^IPV: intimate partner violence.

^c^Two of the women reported experiencing IPV more than 1 year prior to their current pregnancy.

Of the 23 home visitors, 9 were from urban sites and 14 were from rural sites. Some home visitors disclosed their age, while others preferred to select an age band. The age range was between 25 and 66 years. Length of time practicing as a home visitor was between 6 months and 20 years.

Nonparticipant observations were conducted with 4 African American women, 1 aged 21 years, 2 aged 20 years, and 1 aged 35 years. These observations were facilitated by their home visitor, also African American, who preferred not to disclose her age.

### Themes From the Interviews

The relationship between the home visitor and the client is central to the care the home visitor provides and the foundation for promoting positive parenting behavior. The first set of results focuses on key aspects of the relationship that affected the experience of IPV screening, in order to enhance our understanding of how the introduction of the computer tablet transformed these experiences either negatively or positively.

#### The Pivotal Role of the Home Visitor-Client Relationship

The close bond that home visitors developed with mothers was a key factor in engaging them in home visiting activities and bringing about meaningful change in parenting behavior. It was also regarded as essential to facilitating discussion of IPV.

[The most important aspect of home visiting work] is building the relationship because if you don’t have the relationship then you don’t have anything to work with.

Home visitor, ≥46 years, rural

For some women the relationship seemed to replicate familiar bonds of connectedness, which was reflected in their descriptions of their home visitor as being like “a mother figure” or a “close friend.” This was also apparent in the nonparticipant observations of home visits, in which interactions and exchanges were warm and caring in nature. For instance, Tina, a home visitor, would bring her clients clothes, toys, and books from the donations that the home visiting team received. The close bond was expressed directly by women and emphasized the importance of interpersonal communication.

I have confidence in her as a person. She’s a very nice person and caring and you feel affection for her quickly.

Carolina, client, 33 years, rural, IPV–

Regardless of how DOVE was administered, there was concern among home visitors that asking about IPV might damage the relationship they had carefully built with women and that they could potentially lose them from the home visiting program. Their desire to support women, while not wanting to intrude into their personal lives, posed a dilemma for some.

There’s always the concern, you know, will the family or the woman of the household feel like you’re being too invasive and then want to pull away from the program?

Coleen, home visitor, 27 years, rural

Women regarded IPV screening as an opportunity to talk to someone other than family and friends, whose advice might be unwelcome. It made them feel “cared for” that someone wanted to know if they were “going through a hard time.” Furthermore, the screening helped to raise awareness about and destigmatize IPV, thereby making it “more of a common thing” to talk about. This view was shared by women in rural and urban locations, by older and younger women, and among the 3 Spanish-speaking women.

DOVE really helped a lot…Some women could tell you right off the bat “look he beat me.” But some women could be just like me and it takes time. I think if they do it and the home visitor comes in and they’re graceful and supportive, I think it will help [women] a lot. I feel like it helped me a lot and to trust people again.

Joanne, client, 21 years, rural, IPV+

#### Waiting for the Right Moment in the Relationship

Feeling trust in the home visitor facilitated disclosure of IPV, and women’s comments emphasized the cognitive and affective aspects of interpersonal trust. Trust was cultivated through repeated interactions, and women assessed trustworthiness on many dimensions, including prior experience or knowledge of the home visitor, the home visitor’s tone of voice, not feeling pressured to discuss details of the abuse, reassurances of confidentiality, belief in the home visitor’s intentions as genuinely caring, their ability to listen, and not appearing to be uncomfortable with the issue. Women also talked about trust based on “instincts,” “vibes that I can read off of somebody,” or whether their home visitor’s demeanor resembled that of someone else they had trusted in the past. Ostensibly, women’s disclosure of IPV was a staged process whereby they assessed their home visitor’s reactions before sharing more information about the abuse.

It takes me a long time to trust somebody. When Rachel [home visitor] first started coming here I didn’t like her. I didn’t like talking or anybody messing with my daughter. I didn’t like people talking to me about past things. But she was very graceful with it. She didn’t rush me to want to talk to her. She did it at my own speed and that made me know that she cared…She would ask “what was the worst part about being with Jason?” and she said “you know you don’t have to go into detail, if you can just give me a brief summary, it’ll help out a lot.” She wasn’t all in your face and she had a soft spoken voice where I felt very comfortable.

Joanne, client, 18 years, rural, IPV+

Women assigned to the computer tablet for screening were not obliged to share their answers with their home visitor immediately, although the home visitor would later be informed by the research team if a woman had screened positive and therefore was eligible to receive the DOVE intervention. In the following quote, a woman reflects on the advantage of the computer tablet compared with the home visitor method when trust has not yet been established.

If I did not trust her I would not have done this…if that were the case then I would like the tablet. Then I could have answered the questions and I don’t have to worry if she had seen them.

Kimberley, client, 20 years, rural, IPV+

Home visitors felt that, regardless of the method used, finding the right moment in the relationship to introduce DOVE affected women’s willingness to engage with it. A period of trust and rapport building was also necessary for the comfort of home visitors in finding the right moment to introduce IPV using the computer tablet or the in-person method.

R: Did you feel there were any risks? How did you feel putting the information in the computer tablet?

I: No, ’cause when she came she was partly a friend…She knew I was not from here and that I was not familiar with a lot of things. So she said if you need anybody you can talk to me. And she said, okay you can trust me, I’m not going to tell anybody. It’s just me and you, but I can help you. Here are some people you can talk to.

Bernice, client, 20 years, urban, IPV+

I have to make them comfortable with me and that usually takes a couple of visits…even after seeing them for a second time you still haven’t gained their trust. Even if you introduce it, it’s not a topic they want to be discussing right now. Even if there’s nobody home and they can talk, they’ll say “no.”

Hayley, home visitor, 36-45 years, urban

Eye contact is definitely one way [to assess trust]. Where I’ll sit, I think that’s a physical thing that right at the beginning you know they always will sit away from me. Quite far away from me and as the visits progress, eventually they’ll sit beside me. Sometimes I’ll ask permission, “can I sit beside you because we need to look at something together?” It measures a lot of things I think.

Gina, home visitor, 50 years, urban

During the nonparticipant observations of home visits, Tina spoke about the importance of being flexible and engaging with “the woman’s agenda” on the day of the visit, which sometimes required delaying other assessments, including the introduction of DOVE. During the observation of Rhianna’s home visit, her main concern was finding alternative accommodation, as the roof of her mobile home was leaking. It was difficult for Tina to maintain Rhianna’s attention during the child development assessments, yet Tina carefully negotiated their differing agendas in a way that was sensitive to Rhianna’s needs on the day by listening to her concerns.

#### The Role of Technology in Reducing Anticipated Stigma

The computer tablet appeared to offer women a greater sense of anonymity and privacy, thereby encouraging more openness in answering the abuse questions. One home visitor reported that her client did not disclose abuse on a paper-based IPV assessment that was routinely used within the home visiting program, but disclosed multiple types of abuse in the DOVE study using the computer tablet. The potential reasons for this are revealed in the following comment, where a woman discloses that her fear of being judged led her to withhold certain information when her home visitor screened her for IPV.

I: Oh well, she was talking about fights…that made me feel a little uncomfortable. I was unsure whether or not to tell her the truth or just pass on the question.

R: What did you think might happen if you told her the truth?

I: What she may think about me.

Martha, client, 26 years, urban, IPV+

In using the computer tablet in the way it was originally conceived for the DOVE trial (ie, as an alternative to being screened for IPV by a home visitor), women did not have to engage in discussion immediately, and this seemed to reduce their anxiety about a negative reaction as described in the following comments.

There are just some things you feel ashamed saying, no matter how trustworthy that person…And with a computer there’s no emotion…and you can just say whatever you need to say and you won’t feel like you’re being judged…it was like a security blanket.

Lisa, client, 20 years, rural, IPV+

A lot of people don’t like to talk and express themselves so [the computer] brings it more out of a person even if they’re afraid.

Jennifer, client, 30 years, urban, IPV+

Maybe us asking those questions could be the first time it’s ever been brought up. So if they feel safe enough to do it on the tablet, feeling like it’s a little anonymous, it starts to break down those walls and maybe next time they’ll want to talk about it.

Coleen, home visitor, 27 years, rural

Not all women felt ready to discuss the abuse once it was disclosed. This was due, in part, to their fear of things being taken out of their control, feeling vulnerable about the possible consequences of disclosure, or not wanting to discuss abuse that was not current. The computer tablet may have helped limit the extent to which women re-experienced painful memories that can occur through discussion.

Some women don’t like to talk about it because maybe it’s too painful and I think with those women the tablet might be better because they don’t have to verbalize it…When you verbalize it, like it leads to more conversation you know of what happened. And sometimes I think they have to relive what they went through.

Esther, home visitor, age unknown, urban

I: I think the tablet was a good idea because most people got tablets now, it’s convenient. I did it by myself.

R: Did you want to discuss it after?

I: No, I didn’t’ feel like there was nothing really to discuss since I wasn’t going through it at [that] point in time.

Tammy, client, 23 years, urban, IPV+

I saved it [in the computer tablet] and I gave it back to her and she asked me if there was anything I wanted to tell her. I told her a lot, but not all of it. I told her how long it had gone on. I told her that his abuse ended with me losing a baby…I was a little fearful, I was a little scared…It doesn’t matter if it happened a month ago or ten years, when you talk about it, it still kind of brings a little bit of fear in your mind and that’s what I was feeling.

Lisa, client, 20 years, rural, IPV+

#### Impact of Technology on Emotional Connectedness and Disclosure of IPV

The interviews with women and home visitors revealed divergent interpretations of how the DOVE technology was used in practice, resulting in very different accounts regarding its impact on connectedness in the relationship. Although home visitors saw many benefits to using the computer tablet, some were apprehensive about its potential impact on their relationship with women. According to the program designers, a key assumption underpinning the design of the computer tablet was that it would collect more accurate information, as the questions were delivered in a standardized way and the anonymity would encourage disclosure. In the following comment, one of the designers reflected on how the computer tablet might affect interpersonal communication.

You know if in fact relationship building is so crucial…you know my only concern was the client goes off, they complete the forms, they do all the work on the tablet themselves. They hand the tablet back. Would the community worker truly sit and still have communication with that client or would they have let the tablet do all the work for them…would there be a loss in that relationship?

Program designer 02

Home visitors and women talked about the need to convey empathy and compassion when asking about IPV and questioned whether the computer tablet would be an impediment to this.

I don’t like it [the computer tablet] but I’m a fixer and I’m a healer. I’ve heard people want to tell [their] stories over and over because they’re still processing them and so I feel like people need to tell. But that doesn’t mean [the tablet] won’t work for others.

Carol, manager, ≥46 years, rural

It’s cold…it’s just her interacting with a machine. So there’s no sympathy, there’s no condolences. There’s no, I want to say loving interaction. No, um, it’s like no comfort, no support you know.

Shaun, home visitor, 25–35 years, urban

You can let more out [when the home visitor asks] than using the computer. I mean both is fine, but I think you should be able to talk about it instead of using a tablet.

Suzanne, client, 26 years, urban, IPV+

I think I actually would have rather talked to Carol [home visitor] because when you talk to your home visitor you build a relationship with them and you start to get comfortable with them.

Lisa, client, 20 years, rural, IPV+

In the following comment, a woman reflected on the fact that she was unable to explain her responses to the abuse questions in the computer tablet, as she felt there was an element of mutual aggression within her relationship.

I: [The tablet] was easy. It seemed easier than it would have been to actually speaking to somebody. ’Cause when I’m talking with somebody I can ramble on, where with the tablet I could just easily put it in. The only thing that would have maybe made it easier is like if I could explain some of my answers. Like I said, it’s a mixed relationship, there’s [?] from both parties you know, there’s anger and stuff. So to be able to explain that yes, this happened, but it happened this way.

R: Was the computer tablet a helpful way to share your experiences of partner abuse?

I: Well, I mean it kind of varies you know…if you have time to sit down and be able to talk to a person that sometimes helps women better than to do it on the tablet.

Lauren, client, 28 years, rural, IPV+

For some home visitors, the technology appeared to conflict with their philosophy of care, which they described as “a relationship-based program” and “engaging the whole family if they want to be engaged.” This was also evident in the nonparticipant observations of Tina’s home visits, in which she involved parents in conversation about their lives and concerns regarding their children before completing formal assessments. Her observations of parent-child interaction and activity in the home also formed an important part of her evaluations. Together these provided her with a more nuanced understanding of women’s circumstances. For example, Tina described her client Keisha (21 years, rural) as “stable” in terms of having secure accommodation, keeping up with health appointments, and receiving support from grandparents. However, based on her observations, Tina confided that she sensed “underlying negativity” from Keisha toward her baby, an unwanted pregnancy resulting from a short-term relationship. Tina alluded to Keisha’s lack of desire to read to her baby, to encourage talking and crawling, and her proclivity to set goals that focused entirely on her own needs.

The nature of the interpersonal relationship also had implications for how women chose to disclose IPV, which ranged from overt disclosure to subtle hints about abuse, which they elaborated upon during further visits. One home visitor said he relied on “observations, the things that moms tell me, demeanor, attitude…if she’s not herself” as a more nuanced way of assessing for IPV. The DOVE technology eliminated this complex process of waiting for the right moment in the relationship to ask about or disclose abuse, which was advantageous to women in terms of being able to access help quickly. However, it seemed to obscure home visitors’ access to the unspoken clues such as body language, eye contact, tone of voice, and other gestures, which created feelings of redundancy for some. In this respect, the technology was seen a potential barrier to conversations that might provide home visitors with a deeper understanding of their clients’ lives. Some home visitors interpreted women’s use of the earbuds with the computer tablet as a request for privacy, and some home visitors appeared to be reluctant to engage women in a discussion about their responses to the questions.

I: When Stephanie [home visitor] brought the DOVE [computer tablet] to me I was open with her and let [her] know the things that go on in the home. But she never asked me you know what answers I put to the questions or anything.

R: How did you feel about that?

I: Fine. I mean she didn’t ask…If she had asked I definitely would have [told her].

Lauren, client, 28 years, rural, IPV+

If they have the headphones on [while using the computer tablet], sometimes I really wonder you know? You’ve provided information, but there’s been no discussion about it. So to see where mom’s understanding is, I think I struggle with that one.

Stephanie, manager, ≥46 years, rural

There’s something impersonal about that tablet…This is one of the most personal things that you can discuss with a woman…when she bares her soul to you, tells you what’s going on, it’s something that touches your soul. So the impersonalness of the tablet bothered me a bit.

Alyson, home visitor, ≥60 years, urban

In contrast to this perspective, some home visitors felt that the computer tablet helped “open the door” to deeper discussion about abuse and other sensitive issues. This was dependent on the approach that they adopted, women’s willingness to allow their home visitor to participate in the process, and the quality of the relationship. Some women chose to approach the computer tablet as a shared activity and wanted their home visitor to sit with them while they completed the abuse and risk assessments or watched the intervention video.

I think [using the computer tablet] in the home is a good thing because you can actually sit with the person face-to-face, be open to them, and then you can get feedback right away, tell them everything and they can help you. After everything [the home visitor] had to grade it and then she’ll say maybe if you get this answer it means something is wrong [referring to abuse score]. Like if you get a 16 it’s not good.

Bernice, client, 20 years, urban, IPV+

Home visitors’ strategies for maintaining interpersonal connection included asking women whether they wanted to discuss anything after using the computer tablet, suggesting that they review and discuss the abuse assessment scores, or surreptitiously monitoring women’s reactions to the abuse questions for signs of upset. One of the older home visitors revealed that she used her own lack of experience with technology as a way of encouraging young women to open up to her with the computer tablet *(* “it’s like I’m saying you’re really tech savvy with this and it’s sort of like a prop you know. Like we’re going to talk about this, but you get to use this tablet”). One of the program designers described the DOVE technology as a “hybrid intervention” where “there is going to be human interaction if you feel that someone is in distress.”

The two moms [are in] the intervention process and [using the computer tablet] opened up conversations, especially about previous relationships. One of the families, I knew about the violence with the father of her first child. So it’s really opened up and gave us a chance to talk about how her relationship now is different and how the past relationship with violence impacted on her daughter’s life.

Pauline, home visitor, 49 years, rural

R: Would you have been okay to leave [your responses] in the tablet and not talk?

P: No, not at that point. We talked to make sure I was okay and stuff. I like to express myself now…like it helps me more to talk about the domestic violence.

Jennifer, client, 30 years, urban, IPV+

The challenge is how to keep it personal. If [women] answer positive on the tablet and then you just close the tablet and “oh thank you” and put it away then you’ve just told her, all I needed was for you to answer the questions. I’m not really here to help you. You have to say okay so this is how you answered and this is how you scored, let’s talk more about that. The computer can’t do that part, all it can do is take down the information and it’s up to the nurse or home visitor to expand upon it and actually get her the assistance that she needs.

Ann, home visitor, ≥46 years, rural

The following results relate to external factors that had an impact on how home visitors and women integrated the technology into home visits. This includes negotiating safety and confidentiality in the home environment, and computer tablet design features and usability.

#### Computer Tablet Usability and Design Features

It took time for the home visitors to integrate the computer tablet into daily practice, describing their record-keeping procedures as “primarily paper driven.” Women of all ages appeared to be more confident with the technology and mostly asked for help with understanding the questions. Some home visitors felt it was easier to keep track of things with the paper method, and that there was greater risk of something going wrong with the computer tablet.

I think I’m more comfortable with paper. I’d say I’m old fashioned. I think because I know that all I have to do is keep up with it. You know there’s not a chance of something going wrong or something not saving. So I think I feel like I have more control over the paper copies.

Coleen, home visitor, 27 years, rural

I didn’t think much of it ’cause there’s a lot of stuff on tablets nowadays. It’s all getting a little more technological. I really didn’t think anything of it. I thought it was cool.

Tammy, client, 19 years, rural, IPV–

The rural sites were more susceptible to loss of Internet connectivity, which would interrupt the process or delay transfer of information to the university’s server. The program designers reported that updates to the computer tablet were changed from manual to automatic at an early stage of the study, but problems persisted because some home visitors kept their computer tablet switched off when they were not using them. One of the program designers felt that, on reflection, more time should have been included in the training to provide home visitors with “a bit more knowledge of mobile networks and how the Google system works”. Furthermore, they felt that a clean version of the Android system should have been designed for DOVE, as network speed and automatic updates was also affected by bloatware (ie, the preinstalled apps). The trial coordinators were available to deal with computer tablet issues, but it was often necessary to drive long distances to the rural sites to resolve problems. Although a small pilot test was undertaken with clients, the program designers suggested that a more extensive period was needed for end user input during the development and pilot-testing stages.

They were doing [DOVE] then turning [the tablets] off, imagining that the information was being uploaded as it was being done. But because the network is not that good, that wasn’t happening…so the information would stay in the tablets for several days.

Program designer 02

You need to use your tablet often for the tablet to keep connectivity with the Google Play Store and sometimes these tablets sit in a drawer and they miss updates because they’re turned off. They lose the token that Google gives the tablet to keep it authenticated…If you don’t have that token you will not access the market, you cannot get your update. So skipping updates is really bad when you’re dealing with this kind of research software…I would have given them a bit more network knowledge. We didn’t teach them about that…I mean they’re nurses and they’re not supposed to know those things.

Program designer 01

Women and home visitors appreciated some features of the computer tablet; for example, it helped to reduce the cognitive load by presenting women with one question at a time. It also had audio capability, as the DOVE research team anticipated that some of the women would have low literacy, and these features were beneficial to those who experienced difficulties reading long forms. However, one woman who used the computer tablet commented on the relative benefits of using paper assessments, which she felt would have allowed for more considered responses to the questions.

I think I would prefer paper so I can go and look back like when you’re on one question you might [think] “oh well maybe I should have answered that one different.” Because maybe another question helps better explain…I can go back and see is this really how I feel? Instead of the tablet you just get one question at a time and you can’t see them all [together].

Carrie, client, 29 years, rural, IPV+

The computer tablet’s Internet capability allowed for different interpretations of its function for helping women in other areas of their life, providing further evidence of its interpretive flexibility. One home visitor revealed that she downloaded videos of different health topics for use during visits, another used it to access Web-based assessment tools for women who wanted to return to education, and one woman said that she was shown a Web-based video about the prevalence and causes of IPV.

We don’t have tablets usually so I used the tablet to do some personality tests of my clients who wanted to be in school.

Natalia, home visitor, 47 years, rural

She showed me a video on the tablet on the statistics of [domestic violence] and the age range that it normally happens and why it happens. It was like a YouTube video, but was statistics and girls speaking about it and that sort of thing

Joanne, client, 18 years, rural, IPV+

A limiting design feature was the DOVE intervention, which was a prerecorded video of someone presenting the DOVE pamphlet. The program designers explained that the trial design required that the computer tablet replicate a home visitor-led discussion of the pamphlet. Therefore, it was not possible to incorporate any interactive features or algorithms for tailored messaging, beyond those relating to the Danger Assessment scale score, which informed women of their level of risk of lethal violence and prompted them to talk to their home visitor. The video also ensured that the intervention was delivered to women in a consistent manner, as one program designer revealed that there were concerns that with the home visitor method some “weren’t really spending much time and were just handing the brochure over and not really reviewing it with [women].” Some women found the video too long or difficult to absorb, and questioned the need to watch it again on subsequent visits. There was more flexibility to tailor the intervention content to women’s current needs in the home visitor-led discussion. The repetition of the intervention on 6 occasions was based on the assumption that messages needed to be reinforced in order for women to make changes. Home visitors felt that the video was “not engaging” and that administering a static intervention did not reflect women’s changing needs and priorities. One home visitor suggested varying the content and including videos of survivors’ stories, as the home visitor stated that this strategy had been impactful in educating women in the prevention of sudden infant death syndrome. After watching the video once, some women chose not to view it again when it was offered at later visits or skipped the informational section to focus on the Danger Assessment scale, which helped them to reassess their level of risk of homicide.

I guess that video you’re supposed to watch it every time. I thought you should only watch it the first time. ’Cause I’m like why do they want you to watch the same thing? I asked Natalia [home visitor] if I could just skip the video…I’m like how many times am I supposed to watch it?...I guess the [Danger Assessment scale] is useful depending on what you’re dealing with at home. Your answers will change because you’re not always dealing with the same thing at the same period you’re answering those questions…Some of the stuff was informative, like I never thought of stashing away money and that’s the situation I found myself in.

Carrie, client, 29 years, rural, IPV+

I really feel that the tablet could be better used…They could do case studies…there’s something comforting about knowing that there are other women who have experienced the same as you. My client looked at me one time, and she said “how many more times do we have to do this?” I understand that repetition is part of a learning program. But at some point when you see that this client has moved from here to here, there are other things that you can do.

Alyson, home visitor, ≥60 years, urban

Both of the program designers discussed possibilities for future adaptation of DOVE, including the use of shorter educational messages tailored to different levels of risk women were encountering and interactive features.

I would think about what types of risks are actually being assessed there…what levels of risk make a difference and what levels of education are needed for those risks. Then I would create educational vignettes that were specific, short, and tailored to those risks so that I could trigger them when needed…There’s so much more that can be done with imagery than is needed with text…and potentially inputting some interaction within it.

Program designer 02

Despite having reservations about the repetitiveness of the intervention video, participants perceived some aspects of the information presented as being helpful. For example, in the following quote, a woman describes using the cycle-of-violence information to assess her new nonabusive relationship.

I: Was there anything in particular you liked about the video?

R: There is a young lady, she talks about the stages of different things to look out for and what to do…Yeah, I still go over it, the honeymoon stage. I do it with my new partner. Sometimes I think of my past to my new future. In the cycle it talks about, oh I apologize, I love you, I’m going to do this, everything. ’Cause like I said I went through a big trauma…like right now I have real big trust issues. But that was the thing, the cycle that they tell you you’re going through.

Jennifer, client, 30 years, urban, IPV+

#### Safety, Confidentiality, and the Legitimacy of Asking for Time Alone

An advantage of the computer tablet was its built-in safety mechanism, an icon that switched from the DOVE program to a baby video in the case of an unexpected interruption. This safety feature was greatly appreciated because only the home visitor could reactivate DOVE with his or her unique identification number. In addition, if women wanted privacy when using the computer tablet, they could use earbuds. Despite the relative anonymity of the computer tablet, seeing women in a confidential space remained a challenge for home visitors. This was apparent in the observation of Rhianna’s (20 years, rural) home visit, which was conducted in a cramped bedroom with her mother and her mother’s 4 young children present. It was a struggle for Tina to keep her engaged, as there were constant distractions and interruptions. Some of the women were living in mobile homes or small apartments with friends or family where space was lacking, and it was difficult to obtain absolute privacy where a discussion about IPV could take place comfortably. Furthermore, home visitors’ accounts of overbearing partners revealed that it was not unusual for abusers to direct their hostility toward the home visitor.

I have a client who was abused physically, choked while she was pregnant into unconsciousness. And of course I can’t enroll her [in DOVE] because her husband…he’s there for her every move…and she has to arrange her doctor’s appointments when he’s off from work.

Alicia, home visitor, ≥46 years, urban

I’ve had some clients that the abuser is still around and I could only visit during a certain time on a certain day because he would not be around. And that was very uncomfortable for me and I know it was for her because one day he walked in unexpectedly. They kind of hang around usually like in a corner in the kitchen where they can overhear. It’s all a matter of control and intimidation.

Alyson, home visitor, ≥60 years, urban

When asked how they might procure confidential time with women, home visitors suggested strategies such as taking women to their car, and meeting them at the library or obstetrician’s clinic. Regardless of the method used to administer DOVE, women appreciated the home visitor’s reassurances of confidentiality. Concerns about the computer tablet confidentiality were related to information being inadvertently transferred to the wrong people, and there was a perception that information in the tablet might be open to others, while information given to the home visitor would be kept confidential.

Well I kinda had this thought in my head…what if it’s not going to the people they said it’s going to and then he does find me and then I’m screwed…If I were to tell somebody [in person], I think it would go directly to that person or the people that need to know about it. But with the tablet, technology’s kinda finicky sometimes and it has glitches and you don’t really know where it’s going.

Lisa, client, 20 years, rural, IPV+

Everything you put on the computer everyone can see it. It’s probably better letting the home visitor do it because Miss Laura [home visitor] said that if somebody tries to ask her about me, she can’t tell them.

Amy, client, 16 years, rural, IPV+

In summary, although both the computer tablet and home visitor method clearly had benefits for disclosure of abuse, the nature of the relationship between the home visitor and the woman played a role in how they experienced screening for IPV. The malleability of the DOVE technology was dependent upon how home visitors and women chose to interpret its function and role in the care process, and partly due to its design features.

## Discussion

### Principal Findings

Home visitor-led and mHealth approaches to screening women for IPV and offering interventions can be integrated successfully into perinatal home visiting. However, both approaches require good interpersonal skills, and the development of a trusting relationship was an important aspect of ongoing communication and support regardless of the method used to obtain disclosure of IPV. Although the computer tablet was conceived as an alternative to the interpersonal approach to inquiring about IPV, home visitors and women played a role in how the technology was used and gave it new meaning by maintaining interaction. Through their interpretation of its use, some home visitors and women were able to transform the technology from an *impersonal* artifact to a shared activity. Instead of creating distance, the computer tablet became the conduit through which the interpersonal connection between the home visitor and the woman could be deepened. However, others perceived the computer tablet as a barrier to communication and trust building. The DOVE technology appeared to reduce women’s anticipated stigma because they did not worry about negative reactions to their responses, nor did they feel obliged to discuss their responses with the home visitor immediately. Certain design features within the DOVE technology appeared to constrain its interpretive flexibility, such as the didactic intervention video, which home visitors found difficult to tailor to women’s changing circumstances or feelings toward their partner. Since the content was fixed, it was less amenable to alternative ways of using it. Although home visitors and women felt that the video content was helpful, they were less enthusiastic about the way it was delivered and repeated.

### Comparison With Prior Work

The multiple interpretations of the computer tablet reveal an important aspect of the social shaping of the DOVE technology, which can be understood within the social construction of technology (SCOT). From this perspective, technological artifacts are open to multiple interpretations, which influences their development during the embryonic phase and how they are eventually used in practice [35]. A defining feature of the original conception of SCOT is the idea of relevant user groups who can construct radically different meanings of a technology, known as the technology’s interpretive flexibility [36]. However, Orlikowski argued that the “interpretive flexibility of any given technology is not infinite,” as the material characteristics of technology can constrain human action [23]. This appeared to be the case with the didactic DOVE intervention video, which was a limiting feature of its design. It is well documented that abused women are faced with complex decisions and that safety seeking is a gradual process involving multiple steps or strategies [37]. In our study, home visitors and women identified the need for tailored interventions that reflect women’s changing needs. This was also found in an Australia study of a Web-based safety decision aid for women experiencing IPV. The intervention translated aspects of a brief IPV counselling intervention offered by general practitioners into tailored messages, motivational interviewing, and nondirective problem solving into a Web-based format. Women appreciated having an objective assessment of their situation and felt reassured that their concerns were being taken seriously [19]. Outside of the field of violence, Hall and colleagues’ work on the development of cancer support videos articulated the need for video messages to be short and relatable. They emphasized the need to capture the varying concerns and coping strategies of patients at different stages of the illness. This enabled them to create targeted, tailored videos that could reflect a person’s experience during different periods of time [38].

Studies of mHealth technology addressing other sensitive issues, such as safer sex, human immunodeficiency virus (HIV) infection prevention, substance abuse, and depression, have demonstrated its utility in reducing feelings of stigma that can occur during face-to-face counselling [39-42]. This resonates with a clinic-based US study that found that not all women who screened positive for IPV using a computer wanted to share their answers with their health care provider [15]. In our study, the reduced anticipated stigma reported by women using the computer tablet is a positive finding because they were able to avoid the complicated “dance of disclosure” that often occurs when women talk to their health care provider about abuse [43]. Cultural beliefs about IPV can contribute to abused women developing stigmatized identities that focus on victim blaming. In turn, women may internalize these negative beliefs, which can be a barrier to disclosure and help seeking [44]. Disclosure of IPV is often a staged process, and women in this study required time to develop a trusting relationship with their health care provider before divulging detailed information beyond the initial disclosure [45]. Therefore, mHealth technology can facilitate early disclosure and help seeking.

The importance of the relationship between the home visitor and the woman in facilitating behavior change needs careful consideration when infusing technological interventions into perinatal home visiting. Women who experience IPV often feel vulnerable and afraid. Therefore, the necessity of provider empathy and compassion take on added importance because these qualities are basic to good communication and providing a supportive response. Sensitive inquiry for IPV by health professionals followed by a nonjudgmental response can change the perceived acceptability of IPV among women, which is considered a valuable intervention [46]. This raises questions about the extent to which technology can replicate or complement this.

In midwifery, practitioners have expressed concern that technology may be detrimental to client care as it becomes a replacement for human contact. Technology is represented as “other” to the real work of midwives and the more holistic care of being with the woman [47]. Kennedy and Shannon’s exploration of the process of midwifery care revealed how midwives achieved balance between low and high technological environments and perceived themselves as “instruments” of care through their presence with the woman [48]. In our study, some home visitors felt disconnected from women while they used the computer tablet because they were unable to gage their client’s feelings. Similarly, some women using the home visitor method said they appreciated being able to talk to their home visitor about the abuse because it helped them to release and process emotions. However, divergent views emerged, as the DOVE technology was not necessarily an impediment to the interpersonal relationship but facilitated communication about abuse and other sensitive issues. The potential for mHealth to enhance patient-provider communication has been reported elsewhere. In a study of an mHealth HIV/sexually transmitted infection and drug abuse prevention intervention for primary care, adolescents involved in its development suggested the inclusion of a drug use and sexual risk assessment to facilitate difficult conversations with clinicians [49]. Similarly, the inclusion of the Danger Assessment scale in DOVE provided a way for women using the computer tablet method to discuss increased levels of risk of lethal violence with their home visitor. This complemented the discussion of the tailored safety plan that was always initiated by the home visitor.

Home visitors used multiple strategies to infuse IPV screening and the technology into practice, for example, by judging the right moment when trust had been established; monitoring nonverbal communication; approaching the computer tablet as a joint activity and offering to discuss abuse scores; and respecting women’s wishes to use the tablet alone or not discuss their disclosure immediately. Yet, regardless of the method used, it was sometimes necessary for home visitors to prioritize women’s immediate concerns and delay IPV inquiry until a more opportune moment arose. This echoes the work of Jack and colleagues, who stated that client-centered care is central to good practice and that not addressing a client’s immediate concerns may deter her from discussing her experiences of IPV with her home visitor [13]. This emphasizes the need for health practitioners to remain adaptive to the woman and her situation.

Screening for IPV in the home is not without its challenges. In a clinical environment a certain degree of privacy between the practitioner and patient is expected and can also be created. However, negotiating confidential space within the home was challenging, and some home visitors expressed discomfort in requesting this. Home visitors reported feeling vulnerable when entering the homes of clients where there was a known history of risk behaviors such as drug abuse or criminality. While mHealth apps aim to provide access to tailored health information technology and have the potential to alleviate global health burdens, there are concerns about risk to information security and privacy, which have come under scrutiny. This can impede users’ willingness to share information [50]. The DOVE computer tablet offered privacy and included a safety icon that switched the program to a baby video if there were interruptions at home. Yet, regardless of the method used, women still needed explicit reassurance from their home visitor that their participation would remain confidential, particularly from their partner, and that information would not be accessible to others. This emphasizes the important role of home visitors in gaining women’s trust and vouching for the trustworthiness of the technology.

### Strengths and Limitations

The researcher (LJB) was an international visiting fellow who was not involved in the design of the DOVE trial, nor in the training and support of the home visitors. This unique position of “outsider” helped to elicit data that were diverse and rich. While the study revealed several important findings, it was subject to limitations. The study would have benefitted from the inclusion of undocumented migrant women whose opinions on the use of technology to record abuse experiences may have been less favorable due to concerns about personal information being reported to the authorities. At the time of the interviews, more women were randomly assigned to the home visitor method, which resulted in a smaller number of women using the computer tablet in the overall sample. The imbalance among women who had experienced IPV in the year prior to the current pregnancy was smaller (7 versus 11) than among those who had not (1 versus 7). The inclusion of additional computer tablet-using women may have yielded more diverse views, particularly if this occurred at a later stage of the study when home visitors felt more comfortable integrating the technology. Purposive sampling is not free from bias, and interpretation of the findings is limited to the population under study, in this case infant and early-childhood home visiting programs in the United States where there is continuity of the care provider.

### Conclusions

The DOVE computer tablet was introduced into a nonclinical setting in which the home visitor and the woman could develop a consistent and strong interpersonal relationship. While the computer tablet was sometimes regarded as disruptive to the process of relationship building, it was also perceived as beneficial in opening up communication about a highly sensitive topic. It is important to consider end users and the context into which IPV technology is being embedded to ensure that it complements and enhances the therapeutic relationship. Technological interventions are more likely to be accepted and used if they are underpinned by theory and involve end users during the design and testing phases. An mHealth intervention in perinatal home visiting is an important tool for assisting women in disclosure of IPV, considering help-seeking options, and enhancing their safety. However, this must be accompanied by training to help home visitors successfully integrate the tool into their practice.

## Abbreviations

DOVE: Domestic Violence Enhanced Home Visitation Program
eMOCHA: electronic Mobile Open-source Comprehensive Health Application
HIV: human immunodeficiency virus
IPV: intimate partner violence
IRIS: Internet Resource for Intervention and Safety
SCOT: social construction of technology
